# Design, Fabrication and Characterization of Disk Resonator Gyroscope with Vibration and Shock Resistance

**DOI:** 10.3390/s24237553

**Published:** 2024-11-26

**Authors:** Zhaoyang Zhai, Xiaorui Bie, Bingchen Zhu, Zhenxiang Qi, Bowen Wang, Kunfeng Wang, Xudong Zou

**Affiliations:** 1 The State Key Laboratory of Transducer Technology, Aerospace Information Research Institute, Chinese Academy of Sciences, Beijing 100190, China; 2 School of Electronic, Electrical and Communication Engineering, University of Chinese Academy of Sciences, Beijing 100049, China; 3 QiLu Aerospace Information Research Institute, Jinan 250101, China

**Keywords:** disk resonator gyroscope, structure design, quality factor

## Abstract

This paper presents a comprehensive optimization of an outer frame anchor disk resonator gyroscope (DRG) with enhanced resistance to vibration and shock, achieved by increasing the resonant frequency of the tub and translation modes. Furthermore, the wineglass mode retains a high quality factor, enhancing sensitivity and reducing the angle random walk (ARW). The performance of the proposed DRG is analyzed using dynamic equations, and its structural parameters are optimized through finite element analysis (FEA). The prototype device was fabricated using a two-mask silicon-on-insulator (SOI) process on (100) single-crystal silicon (SCS), which is better suited for complementary metal-oxide–semiconductor (CMOS) integration compared to (111) SCS. Experimental results show an ARW of $0.63{}^{\circ}/\sqrt{h}$ and a bias instability (BI) of 7.7°/h, with no significant performance degradation observed under vibrational environments, indicating potential for tactical-grade performance.

## 1. Introduction

Gyroscopes are inertial sensors used to measure the rotation of objects in space. In recent years, with the rapid advancement of microelectromechanical systems (MEMS) technology, MEMS gyroscopes have found widespread application across various fields, including inertial navigation systems, automotive systems, and consumer electronics. Compared to traditional mechanical gyroscopes, MEMS gyroscopes offer advantages, such as small size, lightweight, low power consumption, low cost, and ease of integration [1,2]. With the expansion of application scenarios, from robot control and navigation to efficient operations in the mineral industry under complex conditions, the performance requirements for MEMS gyroscopes are becoming increasingly stringent.

MEMS gyroscopes measure angular velocity using the Coriolis coupling effect between the modes of the MEMS resonator. An ideal MEMS gyroscope resonator should possess a set of degenerate modes with identical resonant frequencies and quality factors, as well as orthogonal vibration directions, to achieve optimal sensitivity. In recent years, various gyroscope structures have been proposed, including tuning fork gyroscopes (TFG), quad-mass gyroscopes [3,4], bulk acoustic wave gyroscopes (BAW) [5,6], micro hemispherical gyroscopes [7], ring gyroscopes [8,9], and disk resonator gyroscopes (DRG) [10,11]. Due to its fully symmetric structure, ideal degenerate modes, and compatibility with planar fabrication processes, the DRG holds significant potential.

Since Boeing and the Jet Propulsion Laboratory (JPL) first developed the DRG [12], researchers have proposed numerous methods for optimizing resonator structural parameters [13,14], fabrication process [15,16,17], and the control system [18,19] to further enhance its performance. For example, the stiffness-mass decoupled gyroscope significantly improves the resonator’s quality factor and effective mass by suspending the lump mass [10]. The honeycomb-like [20] and cobweb-like [13] DRGs optimize their geometric shapes to minimize modal frequency mismatch caused by non-ideal factors during fabrication. A radially pleated DRG structure offers a higher thermoelastic quality factor and greater vibration amplitude [21]. Ultra-clean epitaxial polycrystalline silicon packages reduce the thermal stress from packaging and ensure a high-vacuum environment [15]. Mode-matched techniques effectively reduce frequency split and enhance sensitivity [22]. These efforts have significantly improved the angular random walk (ARW) and bias instability (BI) of DRG.

Additionally, the DRG’s resistance to environmental vibration and shock is critical for maintaining performance. Mechanical vibration and shock in MEMS gyroscopes can induce output errors and degrade performance. For vibrations present in everyday environments, [23] indicate that the vibration energy of trucks is mainly concentrated in the frequency range of 5–100 Hz, with an RMS value of 1.17 g. This could result in an erroneous output of about 1°/s for the gyroscope [24]. In single-mass gyroscopes, the proof mass vibrates linearly in-plane at a low frequency with a high quality factor, making it particularly susceptible to external accelerations [25]. In contrast, the operational mode of the DRG is less directly influenced by external accelerations [26]. However, the DRG has several parasitic modes sensitive to vibration and shock that could couple with its operational modes. Therefore, one approach to improving the vibration and shock resistance of DRGs is to increase the resonant frequency of the parasitic modes. Similarly, bulk acoustic wave gyroscopes are resistant to vibration and shock for this reason. However, achieving the high aspect ratio poly and single-crystal silicon (HARPSS) process remains a significant challenge [5]. In [27], a DRG operating in a pseudo-extensional mode is proposed. The inner and outer anchor structures increased the resonant frequency of the parasitic modes. Additionally, studies have shown that adjusting structural parameters can alter the mode order of the DRG. Preliminary frequency sweep tests have validated the feasibility of this approach [28]. It does not involve the analysis and characterization of gyroscope performance, such as BI and ARW. Furthermore, in [29], the compatibility between mode order optimization and quality factor design was considered. The study analyzed the effect of different mass suspension schemes and slot configurations on the quality factor. However, the simulation results require further experimental validation. Additionally, changes in structural parameters also lead to changes in the DRG’s sensitivity and noise characteristics, necessitating comprehensive optimization.

This paper systematically presents the design, fabrication, and experimental characterization of an outer frame anchor DRG, offering a systematic reference for optimization. Structural parameters were optimized through finite element analysis (FEA) to achieve a higher quality factor for the operational modes while maximizing the resonant frequency of parasitic modes susceptible to acceleration. Overall, this is achieved by reducing the effective stiffness of the operational modes and increasing the effective stiffness of the parasitic modes. A two-mask process based on (100) single-crystal silicon (SCS) was designed to reduce fabrication complexity and improve fabrication compatibility. The experimental results indicate that the gyroscope has potential for tactical-grade applications, demonstrating an ARW of $0.63{}^{\circ}/\sqrt{h}$ and BI of 7.7°/h. Under vibrations with a maximum peak-to-peak value of approximately 1.5 g and a frequency range of 15–100 Hz, the DRG’s output remains essentially unchanged. The operational principle of the DRG is introduced in Section 2. Section 3 describes the optimization of the structure, followed by the fabrication process in Section 4. Section 5 presents the experimental results, and the conclusions are provided in Section 6.

## 2. Basic Principles of Operation

### 2.1. Dynamic Equation

**Figure 1:**
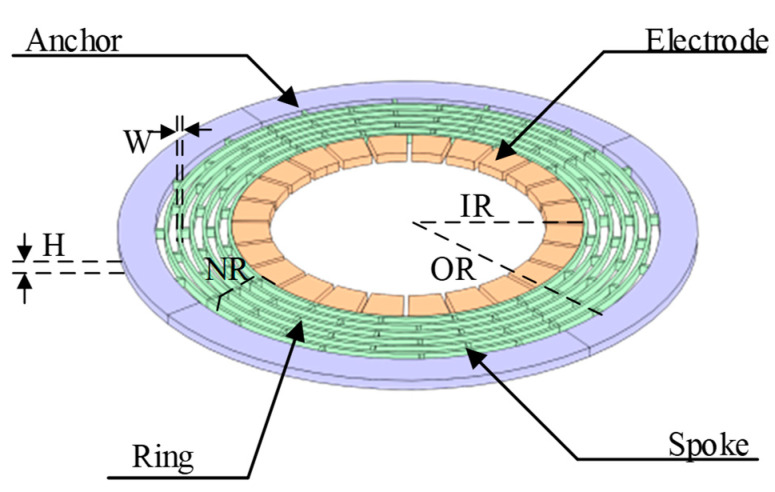
Structure schematic of the DRG.

The structure schematic of the DRG is shown in Figure 1. It consists of an outer frame anchor, concentric rings, connecting spokes and inner electrodes. The basic structural parameters of the DRG include inner ring radius IR, outer ring radius OR, height of the ring H, width of the ring W, and the number of resonant rings NR.

**Figure 2:**
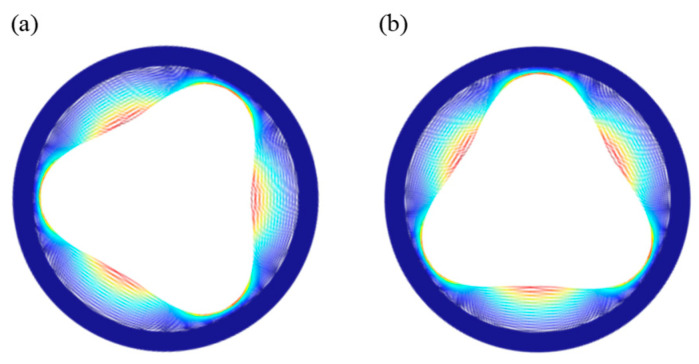
N = 3 wineglass degenerate modes. (**a**) Drive mode, (**b**) sense mode.

Figure 2 shows the vibration modes of the N = 3 wineglass mode of the DRG fabricated in the (100) SCS wafer. The (100) SCS offers several advantages. Due to its high electron mobility, (100) SCS is widely used in the complementary metal-oxide-semiconductor (CMOS), making MEMS devices fabricated on (100) SCS more easily integrated with CMOS circuits [30]. Additionally, unlike (111) SCS, (100) SCS decouples in-plane and out-of-plane deformations, potentially reducing anchor losses [31]. Although its Young’s modulus is anisotropic in-plane, it exhibits 90° rotational symmetry, allowing the N = 3 wineglass modes to achieve equal natural frequencies [32].

The dynamic equation for the DRG is described by two orthogonal second-order systems, which are coupled through the Coriolis force proportional to the applied angular velocity.

(1) $$\left[\begin{array}{cc} M_{11} & 0\\ 0 & M_{22} \end{array}\right]\left[\begin{array}{c} {\ddot{q}}_{1}\\ {\ddot{q}}_{2} \end{array}\right]+\left[\begin{array}{cc} c_{11} & c_{12}\\ c_{21} & c_{22} \end{array}\right]\left[\begin{array}{c} {\dot{q}}_{1}\\ {\dot{q}}_{2} \end{array}\right]+\left[\begin{array}{cc} k_{11} & k_{12}\\ k_{21} & k_{22} \end{array}\right]\left[\begin{array}{c} q_{1}\\ q_{2} \end{array}\right]=\left[\begin{array}{c} F_{1}\\ F_{2} \end{array}\right]+\left[\begin{array}{cc} 0 & 2\gamma \Omega_{z}\\ -2\gamma \Omega_{z} & 0 \end{array}\right]\left[\begin{array}{c} {\dot{q}}_{1}\\ {\dot{q}}_{2} \end{array}\right]$$

The terms $M_{ij}$, $c_{ij}$, and $k_{ij}$ represent the effective mass, damping and stiffness associated with degenerate modes. $q_{1}$ and $q_{2}$ are the displacements of degenerate modes, $F_{1}$ and $F_{2}$ are the drive forces, γ is the Coriolis coupling coefficient, and $\Omega_{z}$ is the applied angular velocity. In a perfectly axisymmetric gyroscope, $M_{11}=M_{22}$, $c_{11}=c_{22}$, $k_{11}=k_{22}$, $c_{21}=c_{21}=k_{12}=k_{21}=0$. When operational in open-loop, Equation (1) can be simplified to:

(2) $${\ddot{q}}_{1}\left(t\right)+{\frac{\omega_{1}}{Q_{1}}\dot{q}}_{1}\left(t\right)+{\omega_{1}^{2}q}_{1}\left(t\right)=\frac{F}{M}$$

(3) $${\ddot{q}}_{2}\left(t\right)+{\frac{\omega_{2}}{Q_{2}}\dot{q}}_{2}\left(t\right)+{\omega_{2}^{2}q}_{2}\left(t\right)=-2nA_{g}\Omega_{z}\left(t\right)\dot{q_{1}}\left(t\right)$$

where $Q_{1}=\omega_{1}M/c_{11}$, $Q_{2}=\omega_{2}M/c_{22}$, $A_{g}=\gamma /nM$, and n is related mode order. Let the drive force be $F=F_{d}sin\omega_{d}t$. Equations (2) and (3) represents damped forced vibrations. The complete solution includes both free vibration and steady-state vibration. Free vibration is a decaying motion that quickly diminishes to zero, so the displacement and phase of the drive mode can be expressed as:

(4) $$q_{1}\left(t\right)=\frac{\frac{F_{d}}{M}}{\sqrt{{\left(\omega_{1}^{2}-\omega_{d}^{2}\right)}^{2}+\omega_{1}^{2}\omega_{d}^{2}/{Q_{1}}^{2}}}\sin\left(\omega_{d}t-\varphi \right)$$

(5) $$\varphi =arctan\frac{\omega_{1}\omega_{d}}{{Q_{1}(\omega }_{1}^{2}-\omega_{d}^{2})}$$

Similarly, the displacement of the sense mode can be expressed as:

(6) $$q_{2}\left(t\right)=\frac{2nA_{g}{\omega_{1}q}_{1}\Omega_{z}}{\sqrt{{\left(\omega_{2}^{2}-\omega_{d}^{2}\right)}^{2}+\omega_{2}^{2}\omega_{d}^{2}/{Q_{2}}^{2}}}\cos\left(\omega_{d}t-\varphi -\theta \right)$$

For the ideal mode-matched DRG, the mechanical sensitivity $S_{mech}$ is represented as:

(7) $$S_{mech}=\frac{2nA_{g}q_{1}Q_{2}}{\omega_{2}}$$

The Brownian motion of the particles inside the resonator generates mechanical-thermal noise. The ARW determined by the mechanical thermal noise is given by:

(8) $${ARW}_{mech}=\frac{1}{nA_{g}q_{1}}\sqrt{\frac{k_{B}T}{M\omega_{1}Q}}\frac{180}{\pi }\times 60{}^{\circ}/\sqrt{h}$$

where $k_{B}$ is Boltzmann’s constant and *T* is the temperature.

### 2.2. Quality Factor

The quality factor is an important parameter affecting the sensitivity and ARW of the gyroscope. It is defined as the ratio of the total energy stored $E_{s}$ to the energy dissipated per cycle $∆E_{d}$, expressed as [33,34]:

(9) $$Q=2\pi \frac{E_{s}}{∆E_{d}}=\frac{energystore}{energydispatedpercycle}$$

The energy dissipated in a MEMS resonator may be influenced by a combination of multiple factors, including thermoelastic damping (TED), anchor loss, air damping, surface loss, and the Akhiezer (AKE) effect. The energy loss caused by each mechanism can be modeled as an individual $Q_{i}$, and collectively influence the total quality factor of the MEMS resonator, which can be expressed as:

(10) $$\frac{1}{Q}=\frac{1}{Q_{TED}}+\frac{1}{Q_{Anchor}}+\frac{1}{Q_{Air}}+\frac{1}{Q_{Surface}}+\frac{1}{Q_{AKE}}$$

TED arises from the coupling between the solid’s strain field and temperature field and has a major influence on the quality factor of the DRG. Anchor loss is caused by the propagation of mechanical waves from the resonator to the substrate. Air damping arises from energy loss due to collisions between the resonator and gas molecules during resonator motion. High vacuum packaging of the DRG can significantly reduce air damping. Surface loss results from the dissipation of energy due to defects, impurities, surface roughness, or other imperfections present on the surface of a resonator. AKE damping originates from phonon interactions, and it can be neglected in low-frequency devices.

Structural parameters have a significant impact on TED. When the resonator deforms, the structural strain induces a temperature gradient, resulting in thermal conduction due to the non-equilibrium temperature field, which leads to the dissipation of part of the mechanical energy in the form of heat. The expression for $Q_{TED}^{-1}$ proposed by Zener is given by [35]:

(11) $$Q_{TED}^{-1}=\frac{E\alpha^{2}T_{0}}{C_{v}}\frac{\omega \tau_{R}}{1+{\left(\omega \tau_{R}\right)}^{2}}$$

where $C_{v}$ represents the material’s specific heat capacity at constant volume, *α* is the thermal expansion coefficient, *E* is the Young’s modulus, ω is resonant frequency, and $T_{0}$ is ambient temperature.

The thermal relaxation time $\tau_{R}$ is:

(12) $$\tau_{R}=\frac{b^{2}C_{v}}{\pi^{2}\kappa }$$

where *b* represents the thickness of the beam where heat flow occurs, and κ is the thermal conductivity. Thermal relaxation time is directly related to the thickness of the resonant beam. When the beam thickness is large, the relaxation time is extended and the vibration period is short, requiring an increase in the resonant frequency to improve the $Q_{TED}$. Conversely, when the beam thickness is small, the relaxation time is reduced, and the vibration period is longer, necessitating a reduction in resonance frequency to enhance the $Q_{TED}$.

### 2.3. Shock and Vibration Rejection

**Figure 3:**
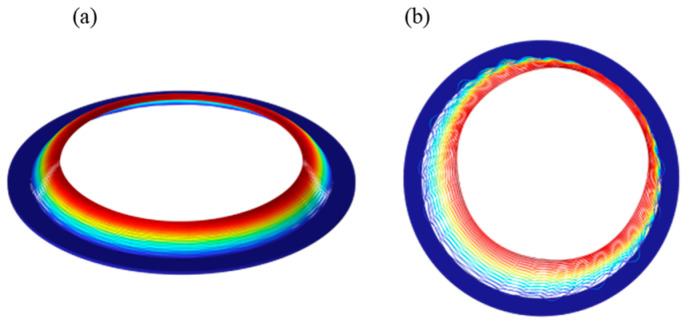
(**a**) Tub mode, (**b**) translation mode.

Compared to traditional mass gyroscopes, the DRG operates in wineglass modes, where the center of mass remains stationary, preventing its operational modes from being driven by external vibrations. However, the DRG still exhibits some parasitic modes, as shown in Figure 3, that vibrate linearly in specific directions, making them susceptible to external accelerations and affecting the output of the sense mode.

**Figure 4:**
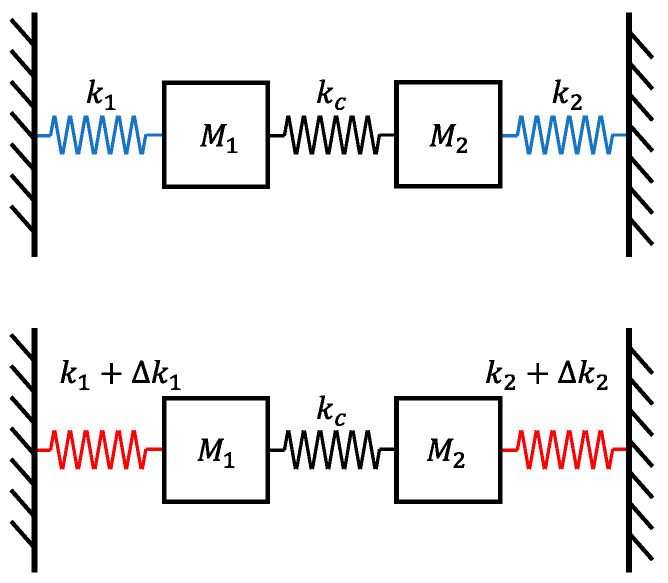
Schematic diagram of a second-order free vibration system.

The DRG’s wineglass and parasitic modes can be approximated by a simple two-degree-of-freedom system as illustrated in Figure 4. In the in-phase mode, two masses vibrate in the same direction. In the out-of-phase mode, the two masses vibrate in the opposite direction. Consider the in-phase mode as the translation mode and the out-of-phase mode as the wineglass mode. However, in reality, the mode shapes of the DRG are significantly more complex.

For resonators 1 and 2 with identical stiffness and mass ($k_{1}=k_{2},M_{1}=M_{2}$), the amplitude ratio between the in-phase and out-of-phase modes is given by:

(13) $$u_{i}=\frac{x_{i2}}{x_{i1}}=\pm 1(i=1,2)$$

The out-of-phase mode cannot be driven by external acceleration. Additionally, the inherent frequency difference results in the vibration response of the in-phase mode being outside the bandwidth of the out-of-phase mode.

In practical situations, a coupled mechanical system cannot have perfectly identical stiffness and mass. When stiffness differences exist within the coupled mechanical system, the amplitude ratio is:

(14) $$u_{i}=\frac{x_{i2}}{x_{i1}}=\frac{∆k\pm \sqrt{∆k^{2}+4k_{c}^{2}}}{2k_{c}}(i=1,2,∆k={∆k}_{2}-{∆k}_{1})$$

In such cases, external acceleration can excite the out-of-phase mode, leading to erroneous angular velocity output from the gyroscope.

Under external acceleration excitation, the vibration equation of parasitic modes of the DRG can be expressed as:

(15) $${\ddot{q}}_{v}\left(t\right)+\frac{\omega_{p}}{Q_{p}}{\dot{q}}_{v}\left(t\right)+{{\omega_{p}}^{2}q}_{v}\left(t\right)=a_{v}sin(\omega_{v}t)$$

where $q_{v}\left(t\right)$ represents the displacement of the vibration mode under external acceleration, $\omega_{p}$ is the resonant frequency of the parasitic mode, $Q_{p}$ is the quality factor of the parasitic mode, $a_{v}$ is the external input acceleration, and $\omega_{v}$ is the frequency of the external input acceleration. An approximate solution can be obtained based on the frequency of the external input acceleration:

(16) $$q_{v}\left(t\right)=\left\{\begin{array}{c} \frac{1}{{\omega_{p}}^{2}}a_{v}\left(t\right)\;\omega_{v}\ll \omega_{p}\\ \frac{Q}{{\omega_{p}}^{2}}a_{v}\left(t\right)\;\omega_{v}=\omega_{p}\\ \frac{1}{{\omega_{v}}^{2}}a_{v}\left(t\right)\;\omega_{v}\gg \omega_{p} \end{array}\right.$$

As the resonant frequency of the parasitic mode increases, its sensitivity to vibration decreases.

## 3. Structural Optimization

Based on the above analysis, it can be concluded that increasing the quality factor of the operational mode improves sensitivity. In this design, we focus on the N = 3 wineglass mode. In some other designs, this could be the N = 2 wineglass mode [29]. Additionally, increasing the resonant frequency of the parasitic modes can enhance resistance to vibration and shock. For the center-supported DRG, parasitic mode resonant frequencies are typically lower than those of the operational modes, making it more susceptible to environmental vibrations [21,36]. In contrast, the out frame-anchor DRG allows for mode adjustment by tuning structural parameters, thereby increasing the resonant frequencies of the parasitic modes, while maintaining a high quality factor for the operational modes. The FEA was used to analyze the relationship between the main structural parameters and performance metrics. Based on FEA, parameters such as the resonant frequency FreqN3 and quality factor $Q_{TED}$ of the operational mode, as well as the resonant frequency FreqTub and FreqTranslation of the parasitic modes, can be directly extracted. Subsequently, mechanical sensitivity $S_{mech}$ and angular random walk ARW can be calculated using Equations (7) and (8).

**Figure 5:**
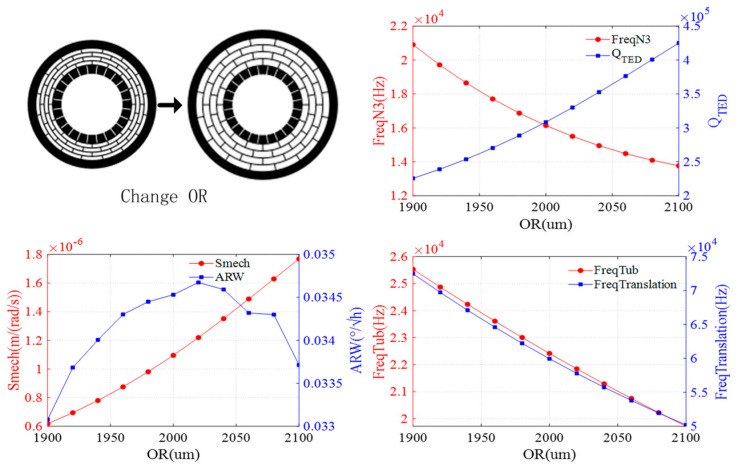
Impact of outer ring radius on DRG.

The effect of the outer ring radius is shown in Figure 5. As the outer ring radius increases, the resonant frequencies of the N = 3 wineglass mode, translation mode, and tub mode gradually decrease, while the $Q_{TED}$ increases. The mechanical sensitivity also increases with a larger outer ring radius. The ARW initially increases and then decreases.

**Figure 6:**
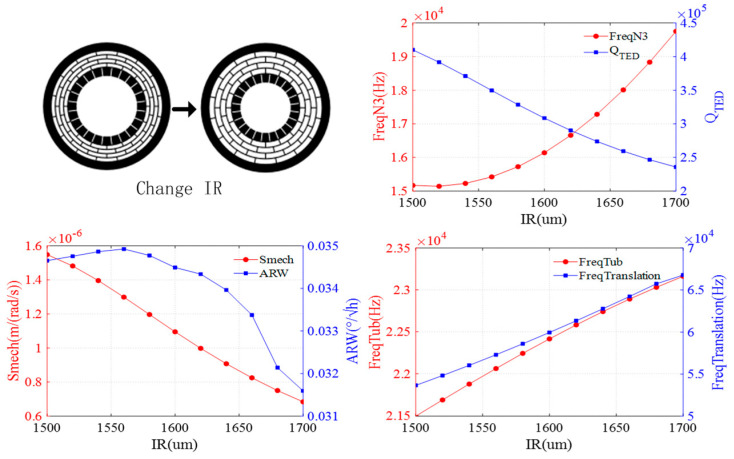
Impact of inner ring radius on DRG.

The effect of the inner ring radius is shown in Figure 6. As the inner ring radius increases, the resonant frequencies of the N = 3 wineglass mode, translation mode, and tub mode gradually increase, while the $Q_{TED}$ decreases. The mechanical sensitivity exhibits a decreasing trend as the inner ring radius increases. The ARW initially increases and then decreases.

**Figure 7:**
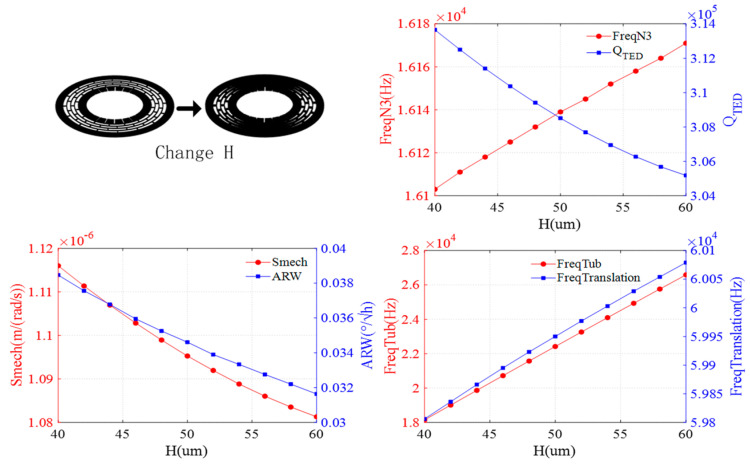
Impact of ring height on DRG.

The effect of the height is shown in Figure 7. As the height of the resonator increases, the resonant frequencies of the N = 3 wineglass and translation modes are almost unchanged, while the resonant frequency of the tub mode gradually increases. Meanwhile, the $Q_{TED}$ and mechanical sensitivity show minimal changes, but the ARW gradually decreases with increasing height.

**Figure 8:**
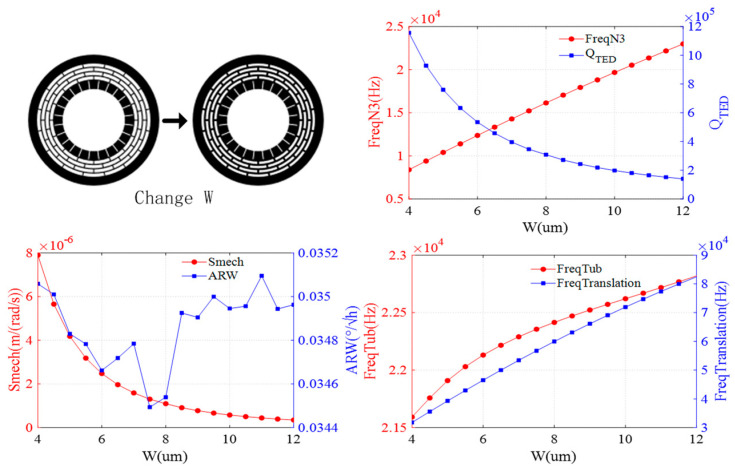
Impact of ring width on DRG.

The effect of the width is shown in Figure 8. As the ring width increases, the resonant frequencies of the N = 3 wineglass, translation, and tub modes increase, while the $Q_{TED}$ decreases. Additionally, mechanical sensitivity decreases. The ARW remains nearly constant.

**Figure 9:**
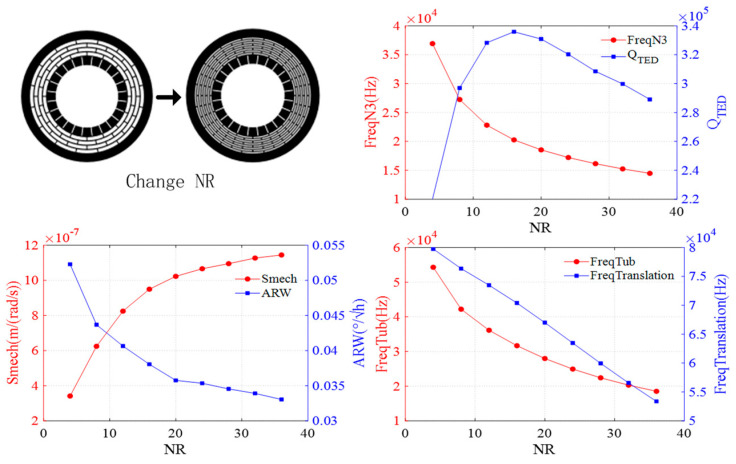
Impact of ring numbers on DRG.

The effect of the number of rings is shown in Figure 9. As the number of rings increases, the resonant frequencies of the N = 3 wineglass mode, translation mode, and tub mode gradually decrease, while the $Q_{TED}$ initially increases and then decreases. Additionally, mechanical sensitivity improves, and ARW decreases.

The final selection of structural parameters must consider fabrication constraints. To ensure uniformity of the etched pattern, the ring thickness should not be less than 6 µm. Due to limitations of the etching aspect ratio, the ring height should not exceed 50 µm. Furthermore, increasing the number of resonant rings complicates the etching process. The final selected structural parameters are listed in Table 1. The operational mode resonant frequency is 16.139 kHz, the in-plane translation mode resonance frequency is 59.950 kHz, and the out-of-plane tub mode resonant frequency is 22.415 kHz. The mechanical sensitivity is $1.095\times 10^{-6}m/(rad/s)$, and the ARW due to mechanical thermal noise is $0.0345{}^{\circ}/\sqrt{h}$. The final structural parameters may not be optimal for a specific performance metric, such as mechanical sensitivity, but they were selected based on a comprehensive consideration of multiple performance factors along with fabrication feasibility.

**Figure 10:**
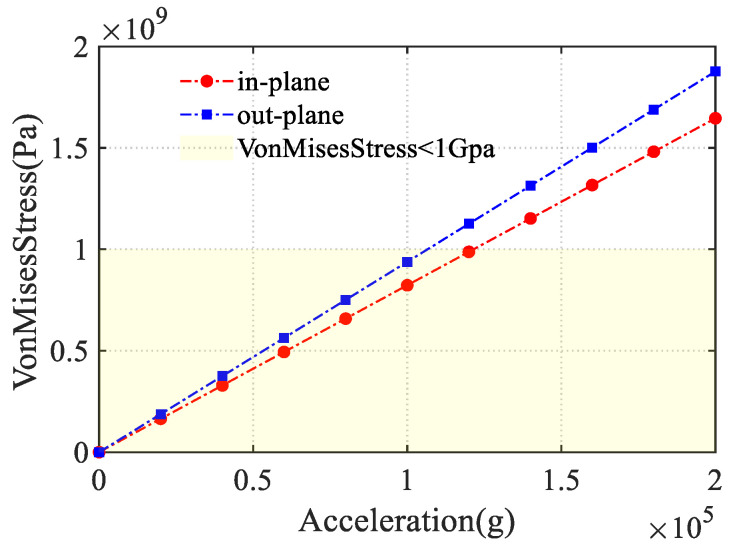
Von Mises stress after applying accelerations.

Then, the simulation of Von Mises stress was conducted using the parameters in Table 1. According to the Von Mises criterion, fracture occurs when the maximum distortion energy density exceeds or equals the distortion energy density at the material’s uniaxial tensile yield point. For the SCS, under the given acceleration, the maximum Von Mises stress is below 1 GPa. Figure 10 shows the overload analysis simulation results for the DRG. Based on the design criteria, the maximum Von Mises stress of 1 GPa corresponds to approximately 120,000 g in-plane and 100,000 g out-of-plane.

**Figure 11:**
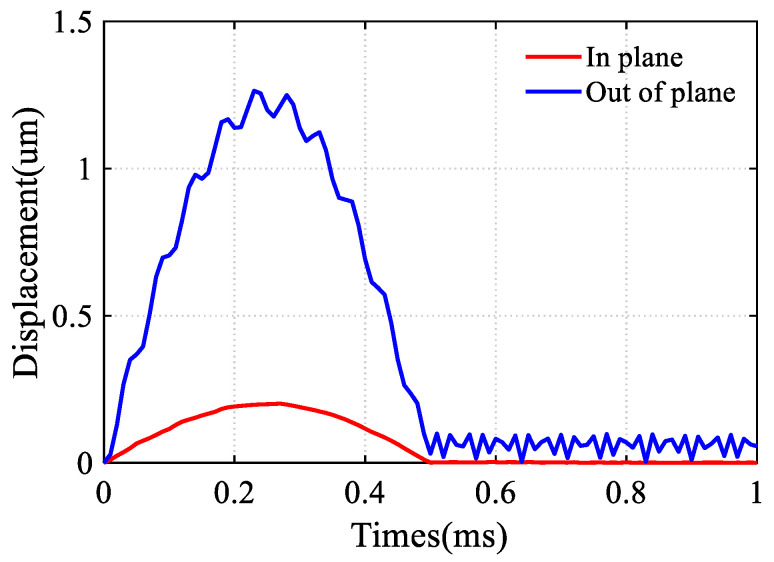
The response of the DRG after applying a 2000 g half-sine shock.

Additionally, shock could potentially lead to the pull-in between the DRG structure and the electrodes, resulting in electrical shorts. The displacement response of the DRG under shock was simulated. A half-sine shock was applied to the DRG, with a shock amplitude of 2000 g and a duration of 0.5 ms. The response curves after applying shocks in both in-plane and out-of-plane directions are presented in Figure 11. The results indicate that the maximum in-plane displacement is 0.2 µm, while the designed capacitive gap is 3 µm. Therefore, electrostatic pull-in is unlikely to occur. Furthermore, the maximum out-of-plane vibration is 1.26 µm. Since the substrate layer and the device layer are at the same potential, the risk of electrical shorts is also eliminated.

**Figure 12:**
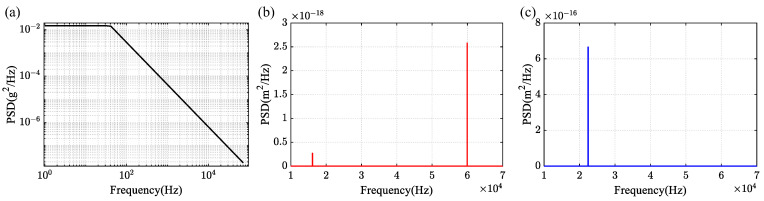
(**a**) The input acceleration power spectral density (PSD). (**b**) The displacement response PSD after applying in-plane vibration. (**c**) The displacement response PSD after applying out-of-plane vibration.

Furthermore, the DRG in random vibration environment was simulated, where the input acceleration power spectral density (PSD) was based on MIL-STD-810H [23] with 1.08$g_{rms}$, as shown in Figure 12a. For in-plane vibration, distinct peaks corresponding to the N = 3 mode and translation mode were observed, as shown in Figure 12b, with an amplitude of $2.66\times 10^{-19}m²/Hz$ at the N = 3 mode. For out-of-plane vibration, the peak corresponding to the tub mode was observed, as shown in Figure 12c. The N = 3 mode exhibited an amplitude $4.39\times 10^{-25}m²/Hz$. The vibration amplitude of the N = 3 mode is consistently smaller compared to that of the translation and tub modes, indicating that the DRG’s operational mode has low sensitivity to linear acceleration. The equivalent calculated g-sensitivity is approximately 0.03°/s/g.

## 4. Fabrication Process

**Figure 13:**
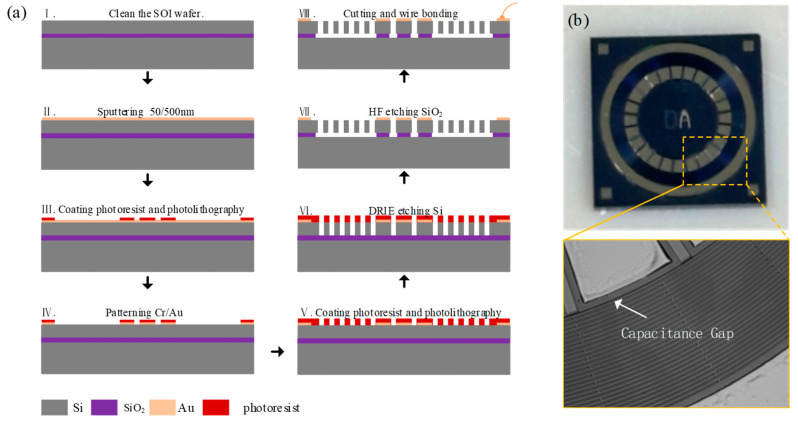
(**a**) The SOI process with two masks. (**b**) The optical image of the prototype device.

The prototype device was fabricated by the SOI process, as shown in Figure 13a. Two masks were used in the process, one for metal layer patterning and the other for patterning the device layer. The detailed process steps are as follows.

First, a 4-inch SOI wafer with 50 μm device layer, 2 μm oxide layer and 300 μm substrate layer was cleaned by a standard wafer cleaning process (see Figure 13a (I)).

Then, the electrodes were fabricated (see Figure 13a (II–IV)). A Cr–Au metal layer with a thickness of 50/500 nm was deposited using magnetron sputtering. Next, the metal layer was coated with photoresist, followed by exposure and development. Using the photoresist as a mask, the metal was etched via metal corrosion to form the electrodes.

Next, the resonator structure was fabricated (see Figure 13a (V–VI)). The residual photoresist from the previous process step was removed, and a new photoresist was coated as the mask for etching the device layer. Then, the resonator structure was etched by deep reactive ion etching (DRIE).

Then, HF vapor was used to etch the 2 μm oxide layer beneath the resonator (see Figure 13a (VII)). After completing the above processes, the devices were cut using a laser cutter, followed by wire bonding for subsequent performance testing (see Figure 13a (VIII)). The optical image of the prototype device is shown in Figure 13b.

## 5. Experiment Results

### 5.1. Readout Circuits

**Figure 14:**
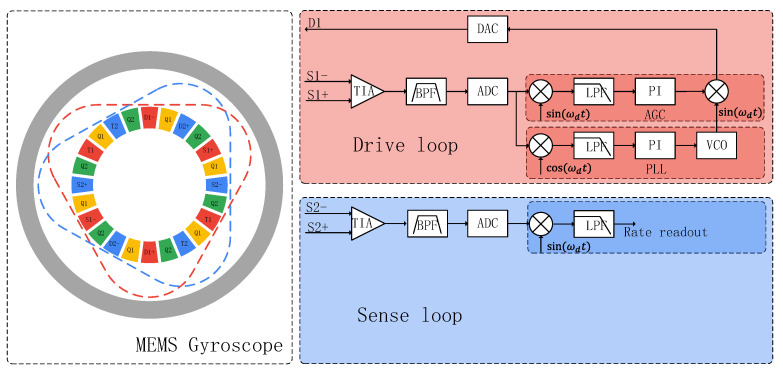
The electrode configuration and measurement circuit of the DRG.

Figure 14 illustrates the electrode configuration and measurement circuit used in the DRG. Electrodes D1 and D2 are the excitation electrodes, used to apply the driving force. Electrodes S1 and S2 are employed for signal readout, while electrodes T1 and T2 are responsible for adjusting frequency split, and electrodes Q1 and Q2 control mode coupling. All of the rings are grounded, with a polarization voltage applied to the drive and sense electrodes. The drive mode operates in closed-loop configuration, with an automatic gain control (AGC) circuit ensuring the stability of the vibration amplitude, while a phase-locked loop (PLL) circuit maintains frequency stability. To minimize circuit noise and improve the signal-to-noise ratio, the front-end analog amplification circuit employs a cascaded structure consisting of a transimpedance amplifier, a differential amplifier, and a band-pass filter.

### 5.2. Performance Characterization

**Figure 15:**
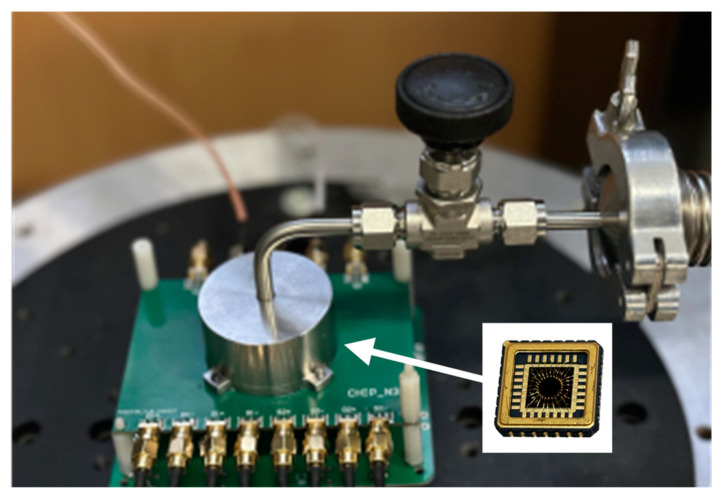
The platform for DRG testing.

The experimental measurements were performed in a custom vacuum chamber equipped with a mechanical pump and a molecular pump. A sealing cap was placed on top of the printed circuit board (PCB) containing the analog front-end amplification circuit, as shown in Figure 15. The vacuum gauge indicated that the pressure inside the chamber reached 0.01 Pa, allowing air damping to be neglected. The DRG was fixed to a leadless chip carrier (LCC28) socket using epoxy resin. The front-end analog PCB was connected to a Zurich Instruments HF2LI lock-in amplifier via SMA interface. The DRG under test was vertically mounted on a uniaxial rate table using screws.

**Figure 16:**
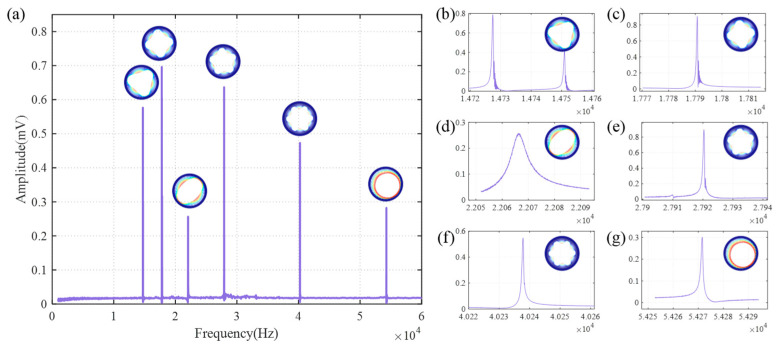
(**a**) Frequency–amplitude response spectrum of the prototype device. (**b**) N = 3 mode, (**c**) N = 4 mode, (**d**) N = 2 mode, (**e**) N = 5 mode, (f) N = 6 mode, (**g**) Translation mode.

The frequency sweep test was performed from 1 kHz to 60 kHz, as shown in Figure 16. The amplitude of each vibration mode may contain errors, partly due to the limited number of sweep points. Additionally, the electrode configuration shown in Figure 14 is specifically designed for the N = 3 mode. In the frequency sweep experiment, D1+ is used for driving, while Q1 in the top right position is used for sensing, which may lead to misalignment between the driving or sensing electrodes and the maximum amplitude of some vibration modes. Nevertheless, this does not affect the characterization of the resonant frequency of each vibration mode. The resonant frequency for the translation mode is 54.27 kHz. The N = 3 wineglass mode has resonant frequencies of 14.73 kHz and 14.75 kHz, both of which are lower than the designed value. This discrepancy is attributed to unavoidable over-etching during fabrication, which led to the actual ring width being smaller than the intended design. The N = 4 mode, which is closest to the N = 3 mode, has a resonant frequency of about 17 kHz. A frequency difference of approximately 3 kHz effectively suppresses the coupling between the modes. The results indicate that, by adjusting structural parameters, higher resonant frequencies of parasitic modes can be achieved.

**Figure 17:**
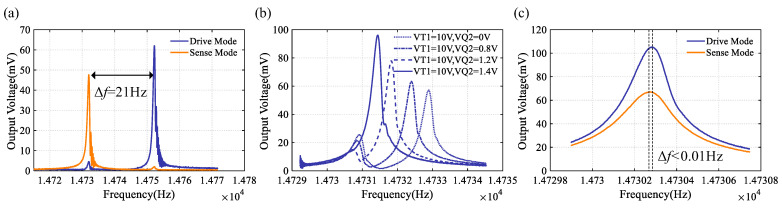
(**a**) The initial frequency response, (**b**) the frequency response during the mode matched process, and (**c**) the frequency response under matched mode conditions.

Although the N = 3 wineglass modes were designed to have identical resonant frequencies, due to process tolerances the fabricated prototype exhibited an initial frequency split of 21 Hz, along with stiffness coupling between the operational modes, as shown in Figure 17a. The electrostatic negative stiffness effect is used to adjust frequency split and stiffness coupling to achieve maximum sensitivity. Figure 17b shows the amplitude–frequency response curve of the drive mode with the VT1 set at 10 V and with VQ2 varying. As VQ2 gradually increases, the amplitude of the resonant peak caused by stiffness coupling decreases, while another amplitude increases, and the resonant frequency decreases, approaching sense mode. As shown in Figure 17c, when VQ2 = 1.44 V and VT1 = 11.45 V, the frequency split of 0.01 Hz can be achieved.

**Figure 18:**
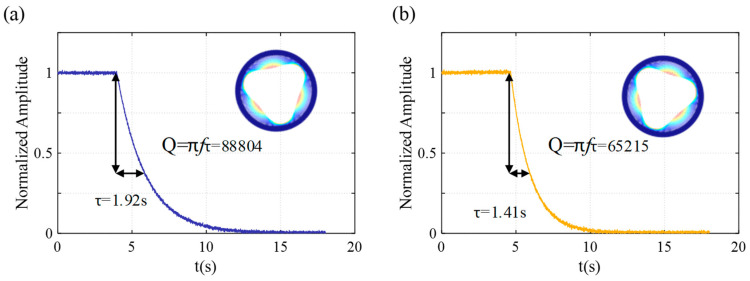
Ring-down quality factor tests. (**a**) Drive mode, (**b**) sense mode.

The ring-down method is used to measure the quality factor of the resonator, as shown in Figure 18. The time constants τ and quality factor for the drive mode are 1.92 s and 88,804, respectively. The time constants τ and quality factor for the sense mode are 1.41 s and 65,215, respectively. The experimentally obtained quality factor is lower than the simulated value, and there is a difference between the drive mode and sense mode. The primary causes are likely to be related to the fabrication process, such as non-uniformity during the HF vapor etching process and low cleanliness of the prototype device surface.

**Figure 19:**
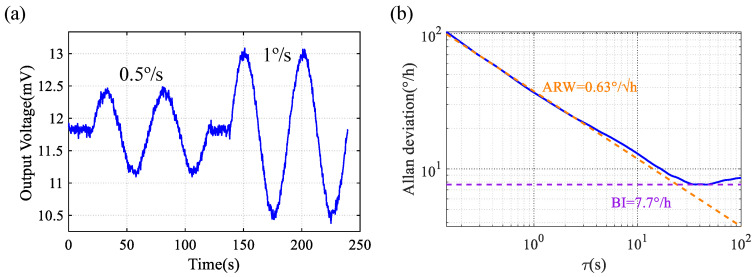
(**a**) Scale factor of this DRG operating in open loop. (**b**) Allan deviation of the DRG.

Under mode-matched conditions, further performance tests were performed. When the turntable outputs sinusoidal angular velocities with peak-to-peak values of 0.5°/s and 1°/s at a frequency of 0.02 Hz, the time-domain waveform of the DRG output signal is shown in Figure 19a. From this, the scale factor of the DRG can be determined as 2.5 mV/(°/s). The performance was tested at room temperature by recording the DRG output without applying any compensation. As shown in Figure 19b, the ARW and BI were $0.63{}^{\circ}/\sqrt{h}$ and 7.7°/h, respectively.

**Figure 20:**
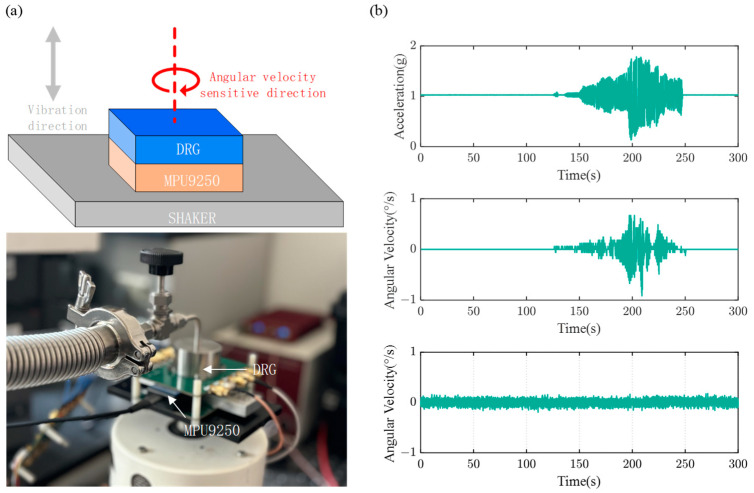
(**a**) Vibration Experimental Setup. (**b**) Test results when vibration direction is parallel to the angular velocity-sensitive axis.

**Figure 21:**
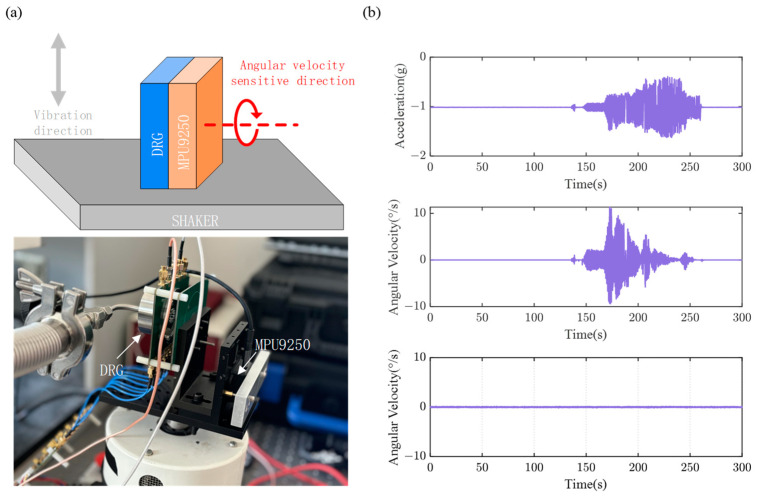
(**a**) Vibration Experimental Setup. (**b**) Test results when the vibration direction is perpendicular to the angular velocity-sensitive axis.

A preliminary vibration test was conducted in accordance with IEEE standard 1431-2004 [37]. Initially, the vibration direction was set to be parallel to the gyroscope’s angular velocity-sensitive axis. The vibration experimental setup is depicted in Figure 20a, where the DRG is mounted on the shaker, with its angular velocity-sensitive axis parallel to the direction of vibration of the shaker. The commercial IMU (MPU9250, InvenSense, CA, USA) was placed back-to-back with the DRG, and a sinusoidal acceleration with a maximum peak-to-peak value of approximately 1.5 g and a frequency range of 15–100 Hz was applied. Simultaneously, the Z-axis accelerometer and Z-axis gyroscope data of the MPU9250, as well as the output of the DRG, were recorded. The results are shown in Figure 20b. Experimental results indicate that, under vibration, the MPU9250 gyroscope produced erroneous output, while the output of the DRG was nearly unaffected. Next, the mounting orientation of both the DRG and MPU9250 was adjusted, as shown in Figure 21a, and the vibration test conducted again. In this case, the vibration direction is perpendicular to the gyroscope’s angular velocity-sensitive axis. The results are shown in Figure 21b. The MPU9250 gyroscope exhibited even greater erroneous output, while the DRG remained nearly unaffected.

Due to the limitations of the current testing conditions, further testing is required to fully evaluate the vibration and shock resistance of the DRG. The current test results only provide preliminary indications of the DRG’s advantages in these areas. The performance comparison with several typical MEMS gyroscope structures is shown in Table 2, indicating that the DRG structure has potential in vibration resistance.

## 6. Conclusions

This paper presents a systematic optimization design of a DRG with enhanced resistance to vibration and shock, aimed at potential tactical-grade applications. The performance parameters of the DRG were analyzed based on dynamic equations, and structural parameters were comprehensively optimized through FEA. The final design ensures a high-quality factor for the operational mode and a high resonant frequency for the parasitic modes. A two-mask SOI process was designed to fabricate the prototype on (100) single-crystal silicon (SCS). Test results indicated an ARW of $0.63{}^{\circ}/\sqrt{h}$ and a BI of 7.7°/h, with no significant performance degradation observed under vibrational environments. These results lay the foundation for further research and the development of high-performance DRGs.

There is still much work to be done in the future. In optimization of the structure, coupling effects between parameters will be considered to avoid local optima. Moreover, the dominant influence of each structural parameter on performance will be explored, and parameter optimization will be conducted based on specific application scenarios. The quality factor of the prototype device differs from the designed value, and further optimization of the fabrication is needed to reduce process tolerances. Additionally, vacuum packaging will be implemented for the prototype to allow for further characterization of its dynamic, vibration, and shock performance. After vacuum packaging, applying higher shock loads is essential for an adequate evaluation of its shock resistance.

## Figures and Tables

**Table 1:** Structural parameters based on finite element analysis.

Structural Parameters	Variable	Value
out ring radius	OR	2000 μm
inner ring radius	IR	1600 μm
ring height	H	50 μm
ring width	W	8 μm
ring number	NR	28

**Table 2:** Performance comparison of typical MEMS gyroscopes.

Affiliation	Type	BI(°/h)	Vibration-Sensitive (°/s/g)
Georgia Institute of Technology [24]	BAW	3.4	0.012
Silicon Sensing [38]	Ring	12	0.17
ADI [24]	TFG	4	0.515
This work	DRG	7.7	<0.23

## Data Availability

Data are contained within the article.

## References

[1] Nemec D., Andel J., Simak V., Hrbcek J. (2023). Homogeneous Sensor Fusion Optimization for Low-Cost Inertial Sensors. Sensors.

[2] Khan Y.A., Imaduddin S., Singh Y.P., Wajid M., Usman M., Abbas M. (2023). Artificial Intelligence Based Approach for Classification of Human Activities Using MEMS Sensors Data. Sensors.

[3] Prikhodko I.P., Nadig S., Gregory J.A., Clark W.A., Judy M.W. Half-a-Month Stable 0.2 Degree-per-Hour Mode-Matched MEMS Gyroscope. Proceedings of the 2017 IEEE International Symposium on Inertial Sensors and Systems (INERTIAL).

[4] Zhang T., Zhou B., Yin P., Chen Z., Zhang R. (2016). Optimal Design of a Center Support Quadruple Mass Gyroscope (CSQMG). Sensors.

[5] Serrano D.E., Zaman M.F., Rahafrooz A., Hrudey P., Lipka R., Younkin D., Nagpal S., Jafri I., Ayazi F. (2016). Substrate-decoupled, bulk-acoustic wave gyroscopes: Design and evaluation of next-generation environmentally robust devices. Microsyst. Nanoeng..

[6] Parajuli M., Sobreviela G., Pandit M., Zhang H., Seshia A.A. (2021). Sub-Deg-per-Hour Edge-Anchored Bulk Acoustic Wave Micromachined Disk Gyroscope. J. Microelectromech. Syst..

[7] Cho J., Singh S., Nagourney T., Woo J.K., Darvishian A., Shiari B., He G., Boyd C., Bentley E., Najafi K. High-Q Navigation-Grade Fused-Silica Micro Birdbath Resonator Gyroscope. Proceedings of the 2021 IEEE Sensors.

[8] Ayazi F., Najafi K. (2001). A HARPSS polysilicon vibrating ring gyroscope. J. Microelectromech. Syst..

[9] Yoon S., Park U., Rhim J., Yang S. (2015). Tactical Grade MEMS Vibrating Ring Gyroscope with High Shock Reliability. Microelectron. Eng..

[10] Zhou X., Xiao D., Li Q., Xu Y., Wang P., Hu Q., Hou Z., Wu Y., Wu X. Stiffness-Mass Decoupled Disk Resonator Gyroscope with Enhanced Performance. Proceedings of the 2018 IEEE Micro Electro Mechanical Systems (MEMS).

[11] Nitzan S., Ahn C.H., Su T.H., Li M., Ng E.J., Wang S., Yang Z.M., Brien G.O., Boser B.E., Kenny T.W. Epitaxially-Encapsulated Polysilicon Disk Resonator Gyroscope. Proceedings of the 2013 IEEE 26th International Conference on Micro Electro Mechanical Systems (MEMS).

[12] Challoner A.D., Ge H.H., Liu J.Y. Boeing Disc Resonator Gyroscope. Proceedings of the 2014 IEEE/ION Position, Location and Navigation Symposium—PLANS 2014.

[13] Fan B., Guo S., Cheng M., Yu L., Zhou M., Hu W., Chen Z., Xu D. (2019). A Novel High-Symmetry Cobweb-Like Disk Resonator Gyroscope. IEEE Sens. J..

[14] Xu Y., Li Q., Wang P., Zhang Y., Zhou X., Yu L., Wu X., Xiao D. (2021). 0.015 Degree-Per-Hour Honeycomb Disk Resonator Gyroscope. IEEE Sens. J..

[15] Senkal D., Askari S., Ahamed M.J., Ng E.J., Hong V., Yang Y., Ahn C.H., Kenny T.W., Shkel A.M. 100K Q-Factor Toroidal Ring Gyroscope Implemented in Wafer-Level Epitaxial Silicon Encapsulation Process. Proceedings of the 2014 IEEE 27th International Conference on Micro Electro Mechanical Systems (MEMS).

[16] Cao H., Liu Y., Kou Z., Zhang Y., Shao X., Gao J., Huang K., Shi Y., Tang J., Shen C. (2019). Design, Fabrication and Experiment of Double U-Beam MEMS Vibration Ring Gyroscope. Micromachines.

[17] Xu P., Si C., He Y., Wei Z., Jia L., Han G., Ning J., Yang F. (2021). A Novel High-Q Dual-Mass MEMS Tuning Fork Gyroscope Based on 3D Wafer-Level Packaging. Sensors.

[18] He C., Xu Y., Wang X., Wu H., Cheng L., Yan G., Huang Q. (2024). Noise Analysis and Suppression Methods for the Front-End Readout Circuit of a Microelectromechanical Systems Gyroscope. Sensors.

[19] Peng Y., Zhao H., Bu F., Yu L. An Automatically Mode-Matched MEMS Gyroscope Based on Phase Characteristics. Proceedings of the Information Technology.

[20] Li Q., Xiao D., Xin Z., Ou F., Wu X. (2017). A Novel Honeycomb-Like Disk Resonant Gyroscope. Proceedings of the 2017 19th International Conference on Solid-State Sensors, Actuators and Microsystems (TRANSDUCERS).

[21] Ren X., Zhou X., Tao Y., Li Q., Wu X., Xiao D. (2021). Radially Pleated Disk Resonator for Gyroscopic Application. J. Microelectromech. Syst..

[22] Ren J., Zhou T., Zhou Y., Li Y., Su Y. (2023). A Real-Time Automatic Mode-Matching Method Based on Phase-Shifted Virtual Coriolis Force for MEMS Disk Resonator Gyroscope. IEEE Sens. J..

[23] (2019). Department of Defense Test Method Standard: Environmental Engineering Considerations and Laboratory Tests.

[24] Serrano D.E., Lipka R., Younkin D., Hrudey P., Tovera J., Rahafrooz A., Zaman M.F., Nagpal S., Jafri I., Ayazi F. Environmentally-Robust High-Performance Tri-Axial Bulk Acoustic Wave Gyroscopes. Proceedings of the 2016 IEEE/ION Position, Location and Navigation Symposium (PLANS).

[25] Yoon S.W., Lee S., Najafi K. (2012). Vibration-induced errors in MEMS tuning fork gyroscopes. Sens. Actuators A Phys..

[26] Yoon S.W., Lee S., Najafi K. (2011). Vibration sensitivity analysis of MEMS vibratory ring gyroscopes. Sens. Actuators A Phys..

[27] Prikhodko I.P., Gregory J.A., Shin D., Kwon R., Kenny T.W., Judy M.W. Pseudo-Extensional Mode MEMS Ring Gyroscope. Proceedings of the 2019 IEEE International Symposium on Inertial Sensors and Systems (INERTIAL).

[28] Efimovskaya A., Wang D., Lin Y.W., Shkel A.M. On Ordering of Fundamental Wineglass Modes in Toroidal Ring Gyroscope. Proceedings of the 2016 IEEE SENSORS.

[29] Qin Z., Ding X., Ge X., Ruan Z., Li H. (2022). A mode order optimized disk resonator gyroscope considering thermoelastic damping. Int. J. Mech. Sci..

[30] Jiang W., Yin H., Zhang Y., Liu Y., Yu W., Xu J., Zhu H. Study of Electron Mobility on Silicon with Different Crystalline Orientations. Proceedings of the 2012 IEEE 11th International Conference on Solid-State and Integrated Circuit Technology.

[31] Zhou X., Xiao D., Li Q., Hu Q., Hou Z., He K., Chen Z., Zhao C., Wu Y., Wu X. (2018). Investigation on the Quality Factor Limit of the (111) Silicon Based Disk Resonator. Micromachines.

[32] Chang C.-O., Chang G.-E., Chou C.-S., Chien W.-T.C., Chen P.-C. (2008). In-plane free vibration of a single-crystal silicon ring. Int. J. Solids Struct..

[33] Foulgoc B.L., Bourouina T., Traon O.L., Bosseboeuf A., Marty F., Breluzeau C., Grandchamp J.-P., Masson S. (2006). Highly decoupled single-crystal silicon resonators: An approach for the intrinsic quality factor. J. Micromech. Microeng..

[34] Hao Z., Erbil A., Ayazi F. (2003). An analytical model for support loss in micromachined beam resonators with in-plane flexural vibrations. Sens. Actuators A Phys..

[35] Zener C. (1937). Internal Friction in Solids. I. Theory of Internal Friction in Reeds. Phys. Rev..

[36] Ahn C.H., Ng E.J., Hong V.A., Yang Y., Lee B.J., Flader I., Kenny T.W. (2015). Mode-Matching of Wineglass Mode Disk Resonator Gyroscope in (100) Single Crystal Silicon. J. Microelectromec. Syst..

[37] (2004). IEEE Standard Specification Format Guide and Test Procedure for Coriolis Vibratory Gyros.

[38] CRM100 Technical Datasheet. https://siliconsensing.com/wp-content/uploads/crm100-00-0100-132_rev_10-datasheet.pdf.

